# Effects of intermittent fasting on HbA1c and weight in insulin versus oral hypoglycemic therapy-treated patients with type 2 diabetes mellitus: a systematic review and meta-analysis

**DOI:** 10.3389/fnut.2026.1699384

**Published:** 2026-01-30

**Authors:** Jana Al Qudah, Newsha Davoudian Beni, Nooran Ibrahim, Salma Khader, Laura Dempsey, Alexandra E. Butler

**Affiliations:** 1 School of Medicine, Royal College of Surgeons in Ireland - Medical University of Bahrain, Busaiteen, Bahrain; 2 Data Science Centre, School of Population Health, RCSI, Dublin, Ireland; 3 Research Department, Royal College of Surgeons in Ireland – Medical University of Bahrain, Busaiteen, Bahrain

**Keywords:** fasting-mimicking diet, glycemic control, insulin, intermittent fasting, oral hypoglycemic agents, time-restricted feeding, type 2 diabetes mellitus, weight loss

## Abstract

**Aim:**

Intermittent fasting (IF) has emerged as a beneficial dietary strategy for managing type 2 diabetes mellitus (T2DM), with improvements in certain indicators of body composition and cardiometabolic health. However, limited research compares the different effects of IF in oral hypoglycemic agents (OHAs) versus insulin-treated patients.

**Methods:**

A comprehensive search was performed across PubMed, Scopus, and Cochrane Library (January 2010–January 2025). Randomized controlled trials (RCTs) involving adults (>18-years) with T2DM undergoing IF, time-restricted feeding (TRF), alternate-day fasting (ARF), fasting-mimicking diets (FMD), and Ramadan fasting were included.

**Results:**

In total, 12 studies (*n* = 1,441 participants) met the inclusion criteria. IF improved glycemic control in both groups, with HbA1c reductions of 0.54% in OHA-users and 2.8% in insulin-users. In the meta-analysis of four eligible trials (*n* = 280), IF produced a significant pooled reduction in HbA1c (−1.85, 95% CI: −2.86 to −0.84), despite substantial heterogeneity (I^2^ = 98.1%). In contrast, IF did not produce a significant change in body weight (−1.45 kg, 95% CI: −5.51 to 2.61; I^2^ = 96.7%). Most studies reported weight loss, with an average body mass index (BMI) reduction of 1.53 kg/m^2^ in OHA users. Among insulin-users, one study reported a significant reduction in weight for the IF group (−4.77 ± 4.99 kg, *p* < 0.001).

**Conclusion:**

IF represents an effective adjuvant therapeutic strategy in T2DM and could be widely employed in clinical practice.

**Systematic review registration:**

Identifier CRD42025650065, https://www.crd.york.ac.uk/PROSPERO/view/CRD42025650065.

## Introduction

Intermittent fasting (IF), defined as the intentional restriction of food intake during specified periods, has been increasingly promoted as a solution for weight loss, diabetes reversal, and improved cardiovascular health. IF regimens can be divided into four subtypes: time-restricted feeding (TRF, eating within a daily time window), alternate-day fasting (ADF, alternating feeding and fasting days), fast mimicking diet (FMD, mimics the effects of a water-only fast), and periodic fasting (e.g., two non-consecutive fasting days per week) (1). These approaches have been associated with improvements in somatic profiles, glycemic control, and cardio-metabolic parameters in both healthy individuals and those with metabolic diseases (2).

One area of interest is the effect of IF on type 2 diabetes mellitus (T2DM), which affects more than 800 million individuals worldwide and has quadrupled in prevalence since 1990 (3). Healthcare expenditure and treatment complexity continue to rise, with the mean number of daily medications used per patient increasing from 3.7 ± 2.8 medications in 2000 to 5.3 ± 3.2 medications in 2020 (4). Action to address these issues is needed, with lifestyle interventions such as IF being explored as a potential non-pharmacological and inexpensive form of management to improve glycemic control and reduce medication dependence.

Clinical trials have evaluated IF as a primary intervention in controlled settings, limiting its impact and the generalizability of findings to “real world” settings, where patients manage T2DM with a variety of medication regimens and face challenges such as polypharmacy and varying levels of glycemic control. Consequently, there is limited evidence as to how IF performs as an adjuvant therapy in clinical practice, specifically with patients receiving antidiabetic medications.

Even in studies including T2DM patients on medication and IF, there is limited comparative research exploring the impacts of IF on insulin versus oral hypoglycemic agent (OHA) regimes. Previous systematic reviews and meta-analyses have reported that IF can improve several cardiometabolic indicators in adults with T2DM (5, 6). However, these reviews evaluate mixed patient populations and do not differentiate outcomes by antihyperglycemic therapy. No prior review has compared IF responses between insulin and OHA-treated individuals, which is the specific focus of this study. This has clinical importance because, by evaluating how IF interacts with different T2DM medications, an objective interpretation of its effects can be obtained, thus guiding safe integration of fasting into diabetes management strategies.

This systematic review evaluates existing evidence for IF as an add-on strategy in patients with T2DM, comparing the outcomes between insulin-treated versus OHA-treated patients, with a focus on the impact on HbA1c, body weight, and other metabolic indicators.

## Materials and methods

This systematic review was conducted following PRISMA guidelines (7). The protocol was registered in the PROSPERO databases (ID: CRD42025650065). The review adhered to PICO (Population, Intervention, Comparison, Outcomes) criteria.

### Data sources and search strategy

PubMed, Scopus, and Cochrane Library databases were searched from January 2010 to January 2025 to ensure the inclusion of robust, up-to-date data. The specific search window was chosen because standardized IF protocols only became widely studied after 2010, making earlier studies less reflective of current clinical practice. The search focused on RCTs. Keywords and Medical Subject Heading (MeSH) terms were chosen to include the population and interventions of interest, accounting for variation in terminology of diabetes and fasting across all literature; hence, MeSH terms were “Diabetes Mellitus, Type 2,” “Hypoglycemic Agents,” “Insulin,” “Fasting,” “Caloric Restriction,” and “Time-Restricted Feeding,” as well as relevant keywords “intermittent fasting,” “fasting-mimicking diet,” “alternate-day fasting,” “Ramadan fasting” to account in terminology across databases. The search was limited to English-language published studies, which introduces language bias by omitting relevant studies published in other languages. References from key articles were reviewed to identify any relevant studies not captured in the initial search (Supplementary File 1).

### Study selection

Rayyan software (Rayyan Systems Inc., 2025. *Web version*. Cambridge, MA, USA: Rayyan Systems Inc.) was used for study selection. Duplicates were removed and results filtered according to eligibility criteria.

#### Inclusion criteria

- Randomized controlled or noninferiority trials
- Study participants had T2DM and aged ≥18 years
- Study examining effects of intermittent fasting, time-restricted fasting, alternate-day fasting, periodic fasting, or fast-mimicking diet interventions
- Study reported at least one primary outcome, such as changes in HbA1c or body mass index
- Study participants taking either insulin or OHAs, but not both simultaneously
- Study reporting Ramadan
  - Ramadan was treated as a subtype of intermittent fasting, having an average of 13–16 h of fasting per day

#### Exclusion criteria

- Non-randomized, observational, case reports, reviews, editorials, abstracts, or protocols
- Participants aged <18 years
- Patients without a confirmed diagnosis of T2DM, or receiving both insulin and OHAs simultaneously
- Use of non-intermittent fasting dietary interventions (e.g., continuous caloric restriction)
- Failure to report relevant outcomes (e.g., HbA1c and fasting glucose)
- Non-English reports

All authors independently screened all citations for title, abstract, and full text. Studies that met the initial inclusion criteria were then assessed through independent full-text screening to confirm eligibility. Discrepancies were discussed collectively to reach a consensus.

**Figure 1:**
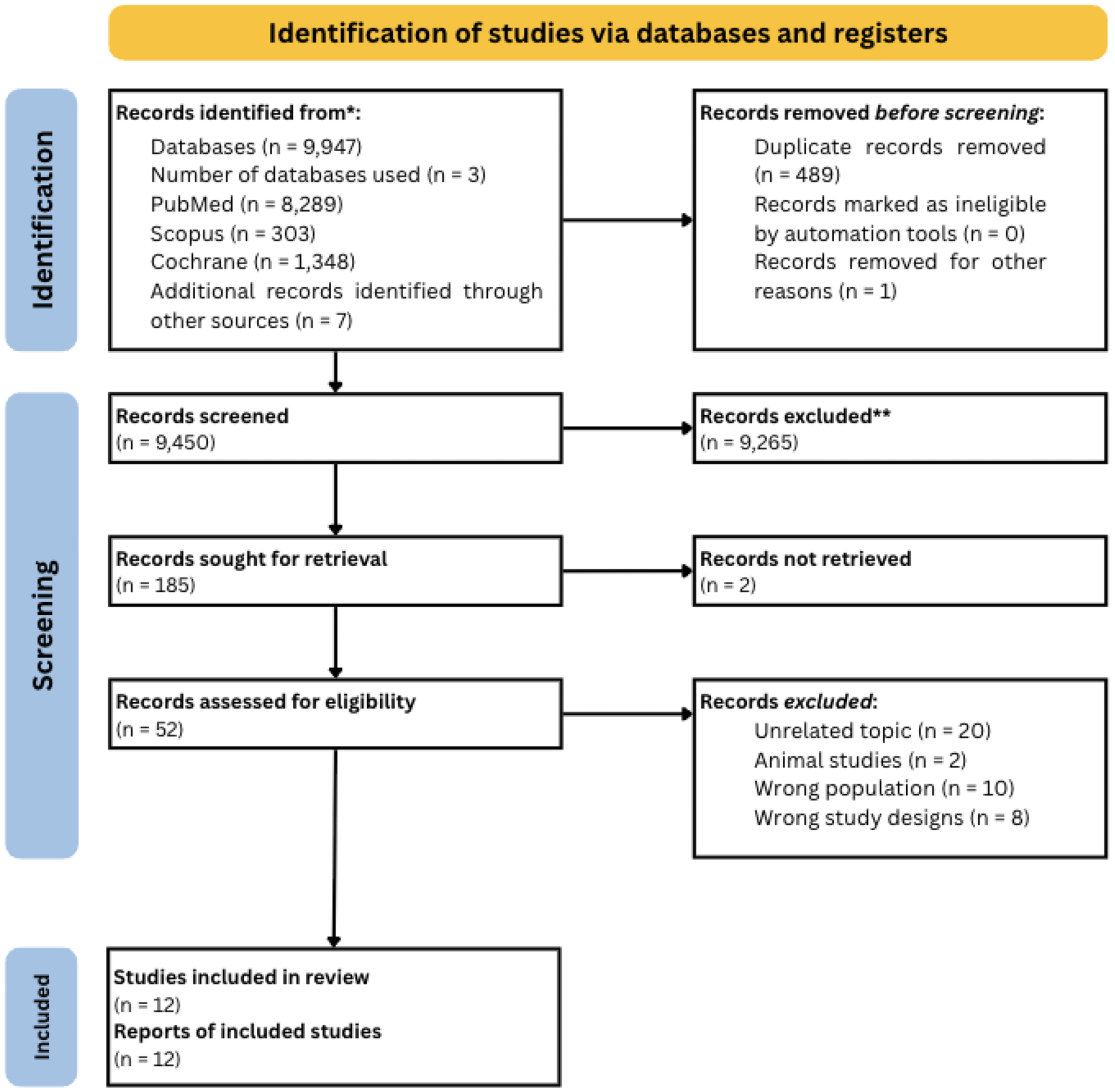
PRISMA flow diagram of study selection. Flowchart illustrating the identification, screening, eligibility, and inclusion process of studies in accordance with PRISMA guidelines. The diagram outlines the number of records identified, screened, excluded (with reasons), and included in the review.

The search yielded 9,947 articles. In total, 489 duplicates were removed, 9,450 articles were screened by title, and 9,265 did not meet the eligibility criteria. A total of 185 articles were retrieved for abstract screening, 52 assessed for eligibility, and 12 in total met the inclusion criteria, as shown in the PRISMA flow diagram (Figure 1).

### Data extraction and quality assessment

Quantitative and qualitative data were extracted based on multiple categories, including numerical values and patient experience. Three authors independently assessed the Risk of Bias (RoB) in included studies using the Cochrane Collaboration tool. When necessary, disagreements were resolved through discussion with a fourth author.

### Data analysis

As a result of substantial heterogeneity in fasting protocols, medication regimens, outcomes, and reporting formats, only four trials contained sufficient data to permit quantitative synthesis (meta-analysis). The remaining studies varied in treatment composition, with two trials not being medication specific and the remaining trials representing insulin or OHA-only cohorts. Hence, meta-analysis by medication type was not feasible.

For the limited number of feasible studies (*n* = 4), a meta-analysis was conducted to estimate the overall effect of IF on HbA1c and body weight. The analyses for both HbA1c % and weight change were conducted using an inverse-variance random-effects model with τ^2^ estimated via restricted maximum likelihood. The remaining comparisons for all included studies (*n* = 12) used a descriptive analytical approach.

In addition, for studies reporting pre- and post-intervention HbA1c, percentage change was calculated to allow comparison across interventions. Mean percentage HbA1c change was then summarized separately for OHA-treated and insulin-treated cohorts; this summary reflects simple averages rather than a pooled statistical estimate. When HbA1c values were reported in mmol/mol, they were converted to percentage units using standard HbA1c conversion equations to maintain consistency across studies. Otherwise, the remaining numerical data were all extracted directly as reported in the RCTs.

Adherence rates were also calculated as the proportion of participants who completed the fasting intervention relative to the number enrolled.

Any reported Standard deviation (SD) values were taken directly from the RCTs; the only SD values calculated were those derived from error bars in graphical figures. All comparisons between insulin- and OHA-treated groups were therefore narrative and descriptive, intended to highlight patterns rather than compute pooled effect estimates.

### Primary and secondary outcomes

This review focused on the effectiveness of IF with main outcomes being HbA1c, weight change [e.g., body mass index (BMI)], adverse events, and patient adherence. Secondary outcomes included fasting blood glucose (FBG), medication reliance, and impact on cardiovascular health.

## Results

### Study range and characteristics

The review included 12 studies with a total of 1,441 participants. Data from each study were extracted as shown in Table 1. Sample sizes ranged from 37 (8) to 343 (9) participants per study, with participants with T2DM aged 18–80 years. The mean duration of studies was 22 weeks. The studies varied in terms of the severity of diabetes in patients, with HbA1c levels ranging from 7 to 10% at baseline. Most participants were overweight, with baseline BMI ranging from 23 to 40 kg/m^2^.

**Table 1:** Data extraction table.

#	Author, year, country	Study design	Age	Duration and follow-up (weeks)	Sample #	Medications	HbA1c	Weight change/BMI	Adverse events
1	Obermayer (3), 2023, Austria	RCT	18–75	12	46	Basal insulin (insulin glargine U300)	HbA1c was 67 ± 11 mmol/mol. 66 ± 10 in the control group and 69 ± 12 in the fasting group. The IF group showed a significant HbA1c reduction (−7.3 ± 12.0 mmol/mol) compared with the control group (0.1 ± 6.1 mmol/mol) over 12 weeks (*p* = 0.012)	Baseline: 34.3 ± 4.5 kg/m^2^ Control: 35.0 ± 4.3 kg/m^2^ Fasting: 33.5 ± 4.7 kg/m^2^	5 serious adverse events leading to hospitalization were reported, 2 in the IF group and 3 in the control group. None of the serious adverse events were considered to be related to the study intervention.
2	Che (16), 2021, China	RCT	18–70	12	104	Control group: OHA 46, insulin 15 TRF group: OHA 42 insulin 19	Control reduction −0.66% ± 0.16 mmol/L [8%]; *p* < 0.001 TRF − 1.54% ± 0.19 mmol/L [18%]	Control group 0.42 ± 0.24 kg/m^2^ [2%], *p* < 0.001 TRF group −1.64 ± 0.38 kg/m^2^ [6%] TRF group: −2.98 kg vs. − 0.83 kg (*p* < 0.001)	1 hypoglycemic event in the control group; no hypoglycemic events in the TRF group. Another study on 8-h TRF reported a nonsignificant increase in the incidence of adverse events, such as nausea, diarrhea, and dizziness
3	Kumar (12), 2024, India	RCT	30–65	26	200	Metformin, sulfonylurea	Initial: 7.0–10.0%. Results: IF group: 8.1 ± 0.8 Control group: 8.0 ± 0.9 *p* = 0.408.	Initial: 23–40 kg/m^2^ Results: IF group 28.9 ± 4.2 kg/m^2^; 4.8 kg decrease Control group: 29.3 ± 4.5 kg/m^2^ *p* = 0.50; 1.5 kg decrease	No severe hypoglycemic events reported; mild hypoglycemia and minor adverse events (nausea, vomiting) are consistent with the safety data from other IF studies.
4	van den Burg (13), 2024, Netherlands	RCT	18–75	52	100	Metformin	Control group: Increased from 53.7 ± 12.2 mmol/mol (7.1 ± 1.1%) to 53.8 ± 7.6 mmol/mol (7.1 ± 0.7%) FMD group: Baseline: 52.2 ± 9.3 mmol/mol (6.9 ± 0.8%) (mean ± SD) Decreased to 49.5 ± 8.2 mmol/mol (6.7 ± 0.8%) at 12 months	BMI ≥ 27 kg/m^2^, −1.2 kg/m^2^; 95% CI: −1.7 to −0.7; *p* < 0.001	8 serious adverse events occurred in 5 FMD participants. No serious adverse events occurred in the control group; none of the serious adverse events were related to the study. Complaints of energy deficit (fatigue, dizziness, and headache) occurred during FMD periods, causing 5 participants to drop out.
5	Pammer (18), 2024, Austria	RCT	18–75	12	41	Insulin therapy	68 ± 12 mmol/mol	34 ± 5 kg/m^2^	No adverse events reported
6	Corley (8), 2018, New Zealand	RCT	>18	12	37	Combination of metformin and/or hypoglycemic agents	64–86 mmol/mol (8.0–10.0%)	30–45 kg/m^2^	53 hypoglycemic events during 84 days of observation affecting 15 participants; reportedly, no significant increase in hypoglycemic events with intermittent fasting
7	Azar (9), 2016, Lebanon	RCT	18–80	33	343	All participants took metformin, with either liraglutide or sulfonylureas	Liraglutide group: 8.3% (SD: 0.94) Sulfonylurea group: 8.2% (SD: 0.91) HbA1c at start of Ramadan: Liraglutide: 7.2% (SD: 1.1), sulfonylurea: 7.8% (SD: 1.1)	Baseline: Liraglutide = 81 ± 17.1 kg Sulfonylurea = 83.1 ± 16 kg	Mild GI symptoms (nausea, vomiting, diarrhea) during treatment initiation 2 serious adverse events with liraglutide (1 gastroenteritis, 1 heel abscess requiring hospitalization); none with sulfonylurea. Hypoglycemia: 36 liraglutide (23.7%), 34 sulfonylureas (20.9%)
8	Umphonsathien (10), 2021, Thailand	RCT	30–60	20	40	Metformin, sulfonylureas	Mean HbA1C level ± SEM was 7.4 ± 1.1%. In the control group, it was 6.9 ± 0.3, 2 days/week intermittent VLCD 7.5 ± 0.3, and 4 days/week intermittent VLCD 7.7 ± 0.3. Control group decreased by −0.2 ± 0.3 *p* = 0.497, 2 days/week intermittent VLCD decreased by −0.8 ± 0.3 *p* = 0.010, −1.2 ± 0.3 *p* < 0.001 in week 10. In week 20, control group decreased by −0.1 ± 0.3 *p* = 0.862, 2 days/week intermittent VLCD decreased −0.7 ± 0.3 *p* = 0.042 and 4 days/week intermittent VLCD decreased by −1.2 ± 0.3 *p* < 0.001	Mean BMI ± SEM was 30.1 ± 5.9 kg/m^2^. Control group BMI was 29.1 ± 1.7 kg/m^2^, 2 days/week intermittent VLCD was 29.9 ± 1.6 kg/m^2^, and 4 days/week intermittent VLCD was 29.9 ± 1.6 kg/m^2^. Week 10, control group BMI decreased by −2.0 ± 0.5 kg/m^2^ *p* < 0.001, 2 days/week intermittent VLCD −2.1 ± 0.4 kg/m^2^ *p* < 0.001, and 4 days/week intermittent VLCD −3.0 ± 0.4 kg/m^2^ *p* < 0.001. Week 20 control group −2.0 ± 0.6 kg/m^2^ *p* < 0.001, 2 days/week intermittent VLCD −2.1 ± 0.5 kg/m^2^ *p* = 0.001, and 4 days/week intermittent VLCD −3.6 ± 0.5 kg/m^2^ *p* < 0.001.	No serious adverse events were encountered.
9	Carter (11), 2018, Australia	RNIT	≥18	52	97	Sulfonylureas, insulin	Baseline to 12 months: significantly reduced in both groups (continuous group: −0.5% [0.2%] vs. intermittent group: −0.3% [0.1%]; *p* = 0.65) At 12 months, baseline > 8% had the greatest mean change (−1.4% [0.2%]; *p* < 0.001), while baseline < 6% had almost no change (−0.03% [0.05%]; *p* = 0.50).	Both groups lost weight between baseline and 3 months, and maintained at 12 months: mean loss from 3 to 12 months—continuous group: 0.4 [0.5] kg, intermittent: −0.2 [0.6] kg. 21 participants from both groups continued losing weight during the 12 months (continuous: −8.4 [1.2] kg, intermittent: −12.5 [1.8] kg)	Hypoglycemia: 8 from the continuous group, 6 from the intermittent group. Hyperglycemia: 3 in the continuous group, 7 in the intermittent group
10	Yang (17), 2023, China	RCT	38–72	12-week intervention, 12 weeks follow-up, 52 weeks follow-up	72	Sulfonylureas, meglitinides, metformin, dipeptidyl peptidase-4 inhibitors, glucagon-like peptide-1 agonists, thiazolidinedione, and insulin	Baseline: similar between the groups, with the CMNT group at 7.63% ± 1.80 and the control group at 7.52% ± 1.30 (*p* = 0.62). 3-month follow-up: The CMNT group experienced a significant reduction to 5.66% ± 0.58, while the control group increased slightly to 7.87% ± 1.63. The reduction in HbA1c was −1.75% ± 1.52 in the CMNT group compared to an increase of 0.37% ± 1.05 in the control group. Adjusted mean difference: −1.06% (95% CI: −1.48 to −0.65) with a highly significant *p*-value of <0.0001. 12-month follow-up: CMNT group sustained at 5.90% ± 0.33, indicating continued remission. The control group did not achieve remission, and their HbA1c values for this time point were not explicitly reported.	Baseline: comparable between groups—CMNT group at 24.23 ± 2.58 kg/m^2^ and the control group at 23.85 ± 2.50 kg/m^2^ (*p* = 0.53, not significant). 3-month follow-up: the CMNT group showed a significant BMI reduction of −2.41 ± 1.00 kg/m^2^, compared to a minimal reduction of −0.18 ± 1.01 kg/m^2^ in the control group. This difference was statistically significant, with a *p*-value of <0.0001.	No serious adverse events were encountered.
11	Lum (15), 2020, Singapore	RCT	≥21	8	97	OHAs, insulin	Mean baseline: 7.8 (0.9)% (62 [9.9] mmol/mol). Intervention group: significant (*p* = 0.018) decrease in HbA1c level of 0.4% (4.4 mmol/mol) from the pre-Ramadan to the post-Ramadan timepoint, whereas the HbA1c decrease in the control group was not significant (*p* = 0.202)	Not available	No self-reported major hypoglycemia events occurred during Ramadan in either group. There were 14 (30.4%) and 15 (29.4%) self-reported minor hypoglycemia incidents in the intervention and control groups, respectively. However, only 1 case in the intervention group and 5 in the control group were confirmed by SMBG readings (<72 mg/dL). Although the difference was not statistically significant, the control group experienced more actual minor hypoglycemic events than the intervention group.
12	Brady (14), 2014, UK	RCT	≥18 (mean: 52)	21	70	Sulfonylureas, liraglutide, metformin	No change in sulfonylurea group from baseline of 7.7% (+0.02%) 0.32% reduction in the liraglutide group. Significant difference in HbA1c was seen at 3 weeks post Ramadan (coefficient −0.30, 95% CI: −0.56 to −0.04)	Baseline: liraglutide 33.0 (7.3) kg/m^2^; sulfonylurea 30.1 (4.3) kg/m^2^ 12 weeks post Ramadan: liraglutide −2.57 kg, sulfonylurea +0.25 kg	2 in the liraglutide group (1 had hyperglycemia, unrelated to study drug, and 1 tingling/numbness in feet, thought to be related to the drug, so dose reduced from 1.2 to 0.6 mg/day) 1 in the sulfonylurea group (generally unwell with non-specific symptoms, not related to the study drug)

IF, intermittent fasting; T2DM, type 2 diabetes mellitus; OHA, oral hypoglycemic agent; RCT, randomized controlled trial; TRF, time-restricted feeding; BMI, body mass index; FMD, fasting-mimicking diet; VLCD, very low-calorie diet; CMNT, continuous medical nutrition therapy; SMBG, self-monitoring of blood glucose.

Key methodological differences included variations in fasting protocols, duration of interventions (12–52 weeks), outcome measures, and type of diabetes medications. Most studies reported HbA1c and weight as primary outcomes; some also assessed lipid profiles, insulin sensitivity, and quality of life (QoL).

The studies included participants from diverse ethnic backgrounds across multiple countries. Studies were conducted in China, India, Thailand, Austria, the Netherlands, Australia, Lebanon, Malaysia, Algeria, South Africa, the UAE, New Zealand, the United Kingdom, and Singapore. Gender distribution varied, with some studies reporting specific numbers: Obermayer et al. (22 women and 24 men) (3), Umphonsathien et al. (29 women, 11 men) (10), and Carter et al. (77 women, 60 men) (11), while others did not specify gender breakdowns.

## Meta-analysis (*n* = 4 studies)

### Change in HbA1c%

**Figure 2:**
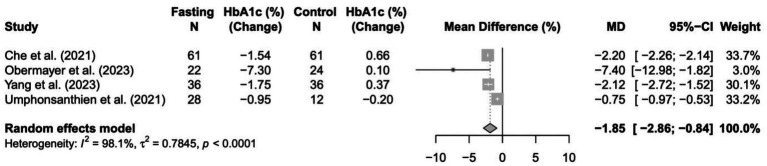
Forest plot of the effects of intermittent fasting (IF) on HbA1c % change in patients with type 2 diabetes.

In a random-effects meta-analysis of four trials (*n* = 280), the fasting intervention produced a statistically significant reduction in HbA1c compared with controls, based on the difference in change from baseline. The pooled mean difference was −1.85% (95% CI: −2.86 to −0.84; *p* = 0.0003), indicating that participants in the intervention groups (fasting) experienced greater decreases in HbA1c than those in the control groups. Substantial heterogeneity was present (I^2^ = 98.1%, *p* < 0.0001), reflecting considerable between-study variability. Results are shown in Figure 2.

### Change in weight (kg)

**Figure 3:**
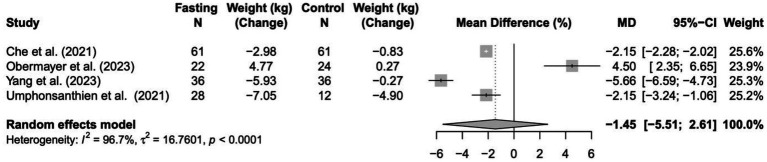
Forest plot of the effects of intermittent fasting (IF) on weight (kg) change in patients with type 2 diabetes.

In a random-effects meta-analysis of four trials (*n* = 280), the intervention did not significantly change body weight compared with controls, based on the difference in change from baseline. The pooled mean difference was −1.45 kg (95% CI: −5.51 to 2.61; *p* = 0.48), indicating no clear evidence that the intervention led to greater weight loss than controls. Heterogeneity between studies was substantial (*I*^2^ = 96.7%, *p* < 0.0001), suggesting considerable variability in findings across trials. Results are shown in Figure 3.

## All included studies (*n* = 12 studies)

### HbA1c reduction

**Figure 4:**
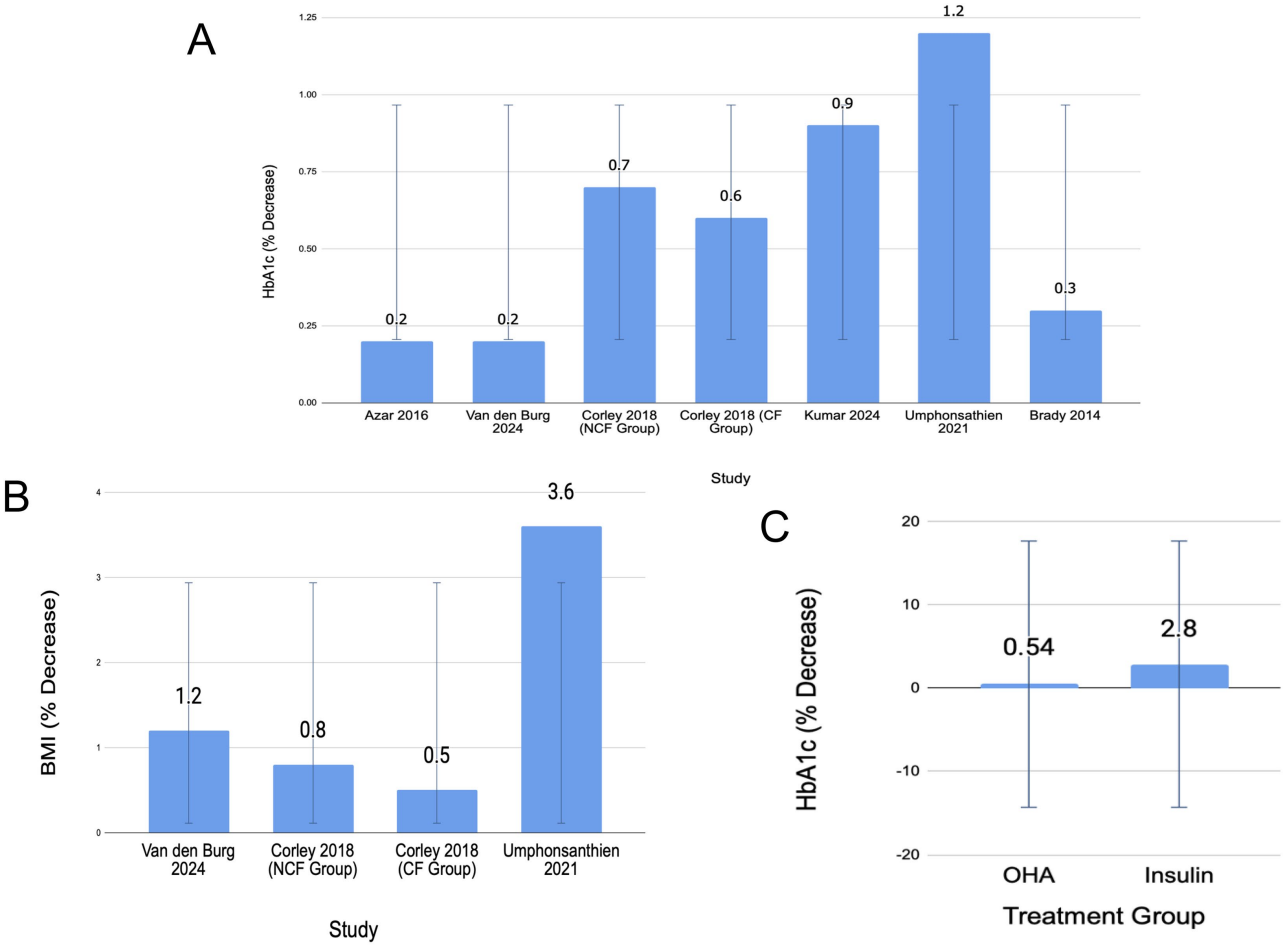
Effects of intermittent fasting (IF) on glycemic control and body composition in patients with type 2 diabetes. **(A)** Mean percentage change in HbA1c across individual studies involving patients treated with oral hypoglycemic agents (OHAs). Error bars represent standard deviation (SD) = 0.38. CF, consecutive fasting; NCF, non-consecutive fasting. **(B)** Comparison of mean percentage change in HbA1c between patients treated with OHAs and those on insulin therapy following IF interventions. Error bars represent SD = 1.59. Data are pooled from eligible studies included in the review. **(C)** Mean percentage change in body mass index (BMI) following IF in patients treated with OHAs. Error bars represent SD = 1.41. Data are derived from eligible studies.

Data were extracted from articles reporting baseline values and post-IF values for HbA1c to calculate percentage change (%). Data for OHAs were retrieved from six investigations (8–10, 12–14) and insulin from one investigation (3). Figure 4A shows the HbA1c% change among OHA medication studies.

The results indicate that IF is beneficial for glycemic control in both groups, with a mean HbA1c reduction of 0.54% in OHA-treated and 2.8% in insulin-treated patients (Figure 4B). IF had a greater impact than insulin with a 2.26 ± 1.59% difference.

Three studies investigated the impact of specific OHA medications under IF conditions (Ramadan, FMD), rather than OHAs as a generalized group (9, 13, 14); thus, we compared the HbA1c reduction in metformin, liraglutide, and sulfonylureas. After 33 weeks, 26.7% of patients treated with liraglutide combined with metformin achieved an outcome of HbA1c < 7.0% in comparison to those treated with sulfonylurea plus metformin; 10.3%, with the study reporting a 0.59% decrease in HbA1c in the liraglutide group and a difference of 0.4% in HbA1c reduction between both treatments while fasting (9). Two studies showed no significant difference in HbA1c using sulfonylureas in contrast to liraglutide. From a baseline of 7.7%, the HbA1c only changed (+0.02%) in the sulfonylurea group, indicating no statistical significance compared to 0.3% in the liraglutide group.

Using metformin only, a study reported a decrease in HbA1c values from 52.2 ± 9.3 mmol/mol (6.9 ± 0.8%) at baseline to 49.5 ± 8.2 mmol/mol (6.7 ± 0.8%) at 52 weeks under FMD interventions (13); 0.2% in HbA1c reduction.

### Weight change

Both OHA and insulin cohorts demonstrated a net weight decrease for patients undergoing either IF, TRF, ARF, FMD, and Ramadan fasting. For the OHA group, three investigations recorded weight change in terms of BMI (kg/m^2^) (Figure 4C) (8, 10, 13). The average BMI change for OHA was −1.53 ± 1.41 kg/m^2^.

Focusing on medication-specific outcomes, sulfonylurea-treated participants experienced a minimal weight increase of 0.34 kg (95% CI: −0.22 to +0.90 kg, *p* = 0.23) over the 33-week study duration. By contrast, the liraglutide group showed a significant weight loss of 3.94 kg (95% CI: −4.54 to −3.33 kg, *p* < 0.0001) at 33 weeks, highlighting the efficacy of liraglutide in promoting weight reduction (12). Similarly, participants in the FMD group taking only metformin experienced an average weight loss of 3.6 kg (95% CI: −5.2 to −2.1 kg, *p* < 0.001) after 12 months (13).

Additionally, two articles reported weight loss of 4.8 ± 3.2 kg in the OHA group (12) and 4.77 ± 4.99 kg in the insulin group, and a decrease in fat mass of 3.5 ± 3.3 kg (BMI not recorded) (3).

### Secondary outcomes

Fasting interventions showed promising effects on metabolic and cardiovascular parameters in T2DM patients in both OHA and insulin cohorts. Several studies reported a reduction in FBG levels; one reported a decrease in mean FBG levels for the pre-Ramadan to the post-Ramadan timepoint at the 3-month follow-up from 123.7 ± 27.1 mg/dL to 122.4 ± 14.5 mg/dL to 120.1 ± 27.1 mg/dL (15). FBG reduction was also reflected by a decrease in the Medication Effect Score (MES) in a TRF study; the MES for OHA and insulin decreased by 19% (change = −0.33 ± 0.27). The same study also hypothesized that, over 12 weeks, TRF results in a significant improvement in glucose regulation and insulin sensitivity in patients taking either insulin or OHA (16).

IF in patients treated with OHAs or insulin had no impact on mean blood pressure (fasting versus non-fasting groups, adjusted mean difference −0.32 mmHg; 95% CI: −3.16 to −2.52; *p* = 0.122) (16). However, the QoL of the fasting group increased by 6.19 ± 6.52, conversely, this decreased by 2.82 ± 3.71, *p* < 0.05 in the non-fasting group at 12-month follow-up (17).

In OHA-treated patients, lipid profiles showed reductions in total cholesterol from 4.2 ± 1.0 to 3.8 ± 0.8 mmol/L (*p* = 0.01) and LDL-cholesterol from 2.1 ± 0.8 to 2.0 ± 0.7 mmol/L (*p* = 0.03) during fasting interventions (8). Triglyceride levels also improved, with one study reporting a decrease from 170.4 mg/dL to 139.3 mg/d (10). Furthermore, one FMD study documented a reduction in MES from 0.7 ± 0.4 to 0.5 ± 0.4 over 12 months; the metformin dose was reduced in 40% of participants and completely stopped in 16% of participants, while among non-fasting patients, only 5% had reduced metformin dosage and 5% stopped the medication (*p* < 0.001) (13).

In insulin-treated patients, IF resulted in a reduction of 9 ± 10 IU in daily insulin dose, while non-fasting patients, by contrast, had an increase of 4 ± 10 IU (*p* = 0.008). This study also reported that IF improves cardiovascular risk factors, baseline HDL, and enhanced cholesterol efflux capacity (3). Furthermore, IF provoked elevation in serum apolipoprotein M (ApoM) levels (*p* = 0.01) (18), which correlated with weight loss and lowering of fasting glucose, suggesting a beneficial impact on cardiovascular health.

### Adverse events

Adverse events were generally mild, with hypoglycemia being most common. Most studies reported no increase in severe hypoglycemia events with IF versus non-fasting groups; however, one study reported 7 patients in the IF group presenting with hypoglycemia (*n* = 51) (11).

Multiple studies reported mild adverse events, mainly gastrointestinal symptoms. One study that included both insulin and OHA medications reported minor adverse outcomes, including fatigue, dizziness, and headaches, during the first week of adaptation (16).

Adverse outcomes differed in OHA versus insulin-treated patients, with a higher incidence of hypoglycemia in insulin-treated groups with consequent insulin dosage reduction required (3).

Two studies reported adverse events specifically in OHA-treated patients. One reported dizziness (*n* = 12), fatigue (*n* = 15), nausea (*n* = 10), and vomiting (*n* = 4) under FMD intervention during its 12-month duration (*n* = 43 completed) (13). Another reported headaches (*n* = 12), dizziness (*n* = 8), and nausea (*n* = 6) in the IF group (*n* = 100) during the intervention’s 6-month duration (12). Insulin-only studies did not report any additional adverse events.

Hypoglycemia incidence varied between OHA treatment groups, with a higher incidence in patients using sulfonylureas versus liraglutide. During Ramadan, 17.8% of patients using sulfonylureas experienced hypoglycemia, compared to 8.6% of those using liraglutide. Documented symptomatic hypoglycemia, such as dizziness or sweating, was also lower (2.0%) in the liraglutide group versus the sulfonylurea group (11.0%). Conversely, mild gastrointestinal symptoms (nausea, vomiting, and diarrhea) were reported in liraglutide users upon initiation of fasting. Major adverse events in the liraglutide group included gastroenteritis (*n* = 1) (9).

### Patient adherence

In total, 8 studies (3, 10–14, 16, 17) reported patient adherence or the number of patients completing the fasting intervention (Table 2). The mean adherence rate was 84.9% in insulin-treated and 84.6% in OHA-treated users, indicating no difference in adherence between groups. Three studies (13, 16, 17) mentioned the cause of dropouts, which were all due to issues with the fasting intervention, such as transportation or lack of contact.

**Table 2:** Baseline HbA1c, HbA1c change (±SD), medication(s) used, study duration, and adherence across studies investigating IF in patients with type 2 diabetes treated with oral hypoglycemic agents (OHAs) or insulin.

Study	Duration (weeks)	Medication(s) used	Baseline HbA1c (%)	HbA1c (%) change	Adherence rate (%)
Van den Burg 2024 (13)	52	Metformin	6.9 ± 0.8	−0.2	84.3
Corley 2018 (8)	12	Metformin/hypoglycemic agent combination	Non-consecutive fasting group: 8.2 ± 1.3	−0.7	N/A
			Consecutive fasting group: 8.4 ± 1.8	−0.6	
Kumar 2024 (12)	24	Metformin, sulfonylureas	8.1 ± 0.8	−0.9	83
Umphonsathien 2021 (10)	20	Metformin, Sulfonylureas	7.4 ± 1.1	−1.2	100
Brady 2014 (14)	4	Liraglutide	7.7	−0.3	70.7
Azar 2016 (9)	33	Sulfonylurea and liraglutide	Sulfonylurea group 7.8	0.21	N/A
			Liraglutide group 7.2	0.2	
Obermayer 2023 (3)	12	Basal insulin (insulin glargine U300)	8.5 ± 3.2	−2.8	91 achieved >75 adherence
Pammer 2024 (18)	12	Insulin therapy	8.4 ± 3.2	N/A	N/A
Carter 2018 (11)	52	Sulfonylureas, insulin	Continuous energy group 7.5 ± 1.4	0.5 ± 0.2%	72.9
			Intermittent energy restriction group 7.2 ± 1.2	−0.3% ± 0.1%	
Yang 2022 (17)	13	Sulfonylureas, meglitinides, metformin, DPP4i, GLP1-RA, thiazolidinedione, and insulin	7.6	0.87	88.9
Che 2021 (16)	12	OHA and insulin	8.68 ± 1.21	1.54 ± 0.19	86.7
Lum 2020 (15)	8	OHAs, insulin	7.8 ± 0.9	0.4	N/A

N/A, not available.

### Study quality

**Figure 5:**
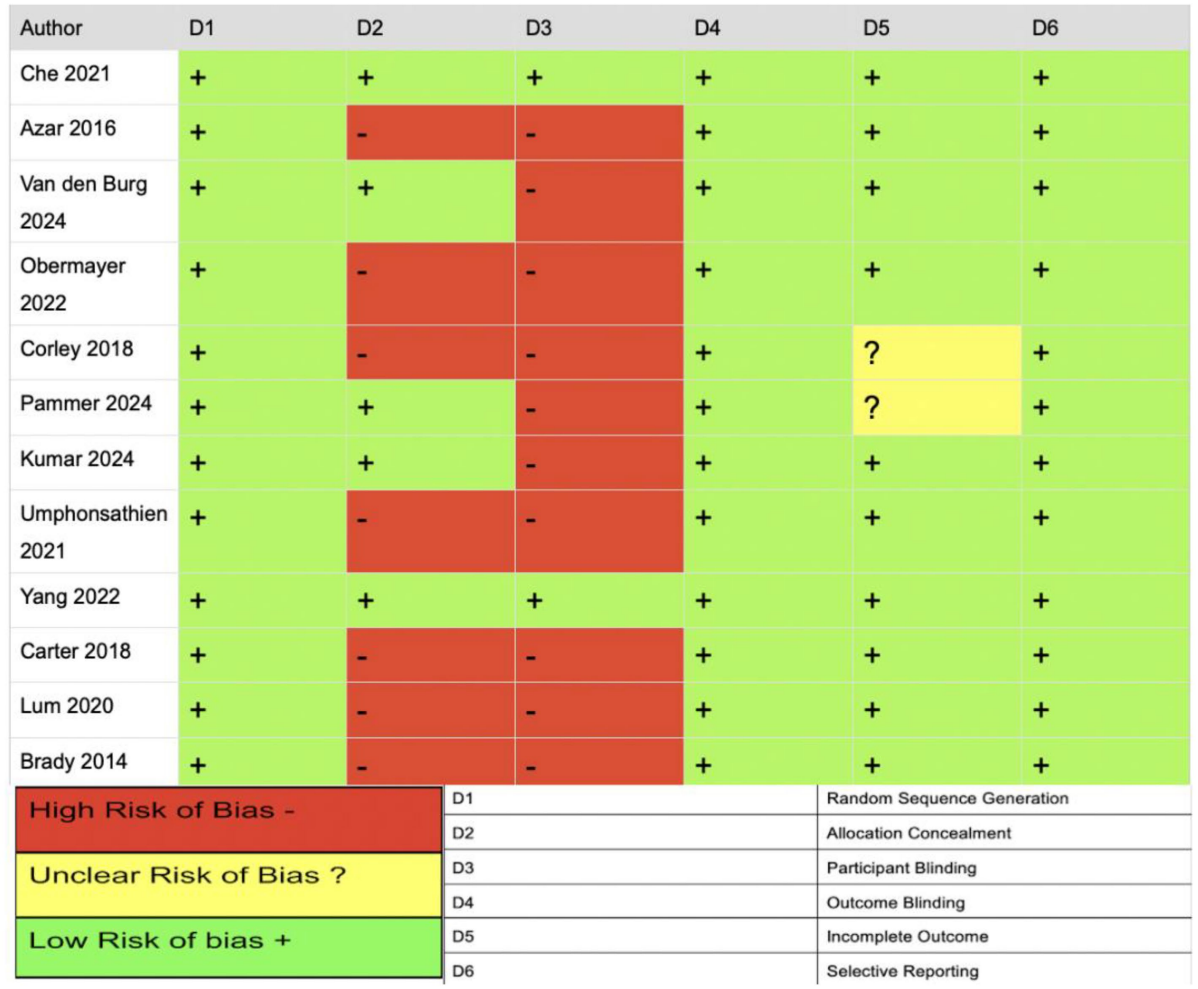
Summary of risk of bias across included studies. Graphical representation of individual risk of bias judgments by domain, using standardized color coding (green = low risk, yellow = unclear risk, red = high risk), assessed via the Cochrane risk of bias tool.

The JBI critical appraisal checklist and Cochrane RoB tool were used to evaluate the studies and indicate that the included studies were generally of moderate quality. RoB was assessed across six domains. The bias risk was low for random sequence generation, indicating that randomization was appropriate and well-described. However, allocation concealment varied. Four studies (12, 13, 16, 18) reported adequate methods (e.g., sealed envelopes), while three studies (3, 8, 9) failed to describe or used inadequate concealment, raising concerns of selection bias. Most studies were high risk for participant blinding; for example, medication regimens (e.g., insulin or OHA) were modified during fasting without standardized protocols or analytical adjustments (e.g., intention-to-treat); this likely introduced performance bias, compromising the internal validity of the intervention effects. For outcome blinding, all studies were rated low risk due to the use of objective endpoints (e.g., HbA1c, glucose, weight, and lipids). However, inclusion of subjective outcomes (e.g., adverse effects and quality of life) without clear assessor blinding introduces the possibility of undetected detection bias. For attrition bias, two studies (8, 18) were rated unclear due to insufficient reporting on dropout rates and imputation methods. Studies were low risk for selection reporting as they appeared to report outcomes aligned with study objectives. However, the absence of registered protocols and inconsistencies in outcome reporting (e.g., selective time points) introduce potential bias (see Figure 5).

## Discussion

The limited meta-analysis that could be undertaken found that intermittent fasting was associated with a significant reduction in HbA1c but did not induce a significant change in body weight when compared with control interventions. Across the four eligible trials, fasting led to a pooled mean decrease of 1.85% in HbA1c, indicating a clinically relevant improvement in glycemic control; however, there was high heterogeneity (I^2^ = 98.1%), reflecting major differences in fasting protocols, participant characteristics, and treatment regimens across studies. In contrast, the pooled effect on body weight was not statistically significant (loss of 1.45 kg; *p* = 0.48) with similarly high heterogeneity (I^2^ = 96.7%), suggesting substantial inconsistency in weight outcomes between trials. Overall, while intermittent fasting may improve HbA1c independently of weight loss, the substantial variability and methodological differences among the included studies indicate that these findings should be interpreted with caution, and firm conclusions about differential effects in insulin- versus OHA-treated populations cannot be drawn due to insufficient data.

Differences in responses to fasting interventions between patients taking OHAs and those taking insulin were found, with a greater decrease in HbA1c in insulin-treated versus OHA-treated patients. However, as only two studies focused on insulin users (3, 18), the data could be skewed or could be consequent upon a higher baseline HbA1c%. For patients using OHAs only, 6 studies (8–10, 12–14) were included, with varying study durations, differences that could have resulted in a lower overall mean reduction. The remaining four studies involved mixed treatment regimens.

When comparing OHA medications, liraglutide combined with metformin demonstrated the greatest HbA1c reduction versus sulfonylurea + metformin and metformin alone over a 52-week period. Liraglutide, a glucagon-like peptide-1 receptor agonist, enhances insulin secretion in accordance with blood glucose, increasing its impact (19). Sulfonylureas, by contrast, stimulate continuous insulin secretion, inducing a moderate HbA1c reduction (20) (greater than metformin, less than liraglutide). Metformin reduces hepatic glucose production, effectively lowering blood glucose but less potently when used as a single therapy (21).

Weight loss and BMI reduction with IF were more apparent in patients taking OHAs, particularly those receiving liraglutide and metformin, agents that more likely enhance weight reduction versus insulin, which counteracts weight loss due to its storage-promoting effects, despite fasting interventions (22, 23). Sulfonylureas, traditionally associated with weight gain, demonstrated no significant weight change during IF (20). These differences underscore the importance of the medication modality in IF.

Fasting interventions are found to improve QoL for patients with T2DM in both OHA and insulin-treated patients, with one article reporting an increase of 6.19 (SD 6.52), *p* < 0.05 at 12 months (17).

There was a reported decrease in FBG in both insulin and OHA patients (15), which was further shown by the decrease in MES score and medication dosage during fasting (16). Fasting also improves glucose regulation and insulin sensitivity (16) by lowering insulin levels, reducing fat stores and inflammation, and promoting metabolic adaptations that increase efficient glucose handling (24). IF had no impact on blood pressure.

Metabolic and cardiovascular responses differed between insulin and OHA-treated patients, likely due to differences in baseline status and medications. OHA-treated patients exhibited more favorable lipid profile changes, such as reductions in total cholesterol, LDL-cholesterol, and triglycerides, as OHAs tend to enhance lipid metabolism, which is amplified by fasting (24, 25).

The reported elevation in ApoM levels in insulin-treated patients suggests an improvement in lipid transport and glucose homeostasis, supporting fasting’s effective role in cardiovascular health. Cardiovascular benefits, including increased HDL-cholesterol, were more pronounced in insulin-treated patients, possibly due to fasting’s role in lowering hyperinsulinemia and inflammation (17).

Insulin secretion and sensitivity are regulated by circadian control and exert significant effects on glucose metabolism; therefore, altered meal timings can further exacerbate glucose intolerance in T2DM patients (26). IF strengthens circadian rhythm alignment, which is a key driver in metabolic improvements and increased insulin sensitivity in T2DM. Fasting creates a consolidated daily feeding window that reinforces the natural day-to-night oscillation of metabolic pathways, restoring synchrony between peripheral clocks in the liver, pancreas, muscle, and adipose tissue. This alignment enhances daytime insulin sensitivity, reduces nocturnal hepatic glucose output, and improves the rhythmic secretion of insulin (27). By re-establishing these circadian patterns, fasting reduces glycemic variability, promotes more efficient glucose handling, and supports better overall metabolic homeostasis in both OHA- and insulin-treated patients.

Adverse events such as vomiting, nausea, headache, diarrhea, and thirst were reported during the first week of IF in both insulin- and OHA-treated patients (16).

Hypoglycemia incidence was higher in insulin-treated versus OHA-treated patients (28). Although no severe episodes occurred, close monitoring and decreased insulin dosage likely mitigated this risk (3). Insulin poses a greater risk of hypoglycemia because it is a direct hormone that immediately stimulates glucose uptake, leading to a steeper drop in blood glucose in comparison to OHAs, which are metabolized more slowly (6).

Although there is a greater risk of hypoglycemia in insulin-treated patients, guidelines are available to increase the safety of fasting. First, insulin dosages should be constantly monitored and eventually decreased, an advantage of IF interventions. Patients should also maintain hydration and balanced nutrition during non-fasting periods, avoid vigorous activity toward the end of the fast, and receive education on recognizing and managing symptoms. Moreover, it is highly recommended to use Self-monitoring of blood glucose (SMBG) devices between 2–5 times a day for T2DM patients to make sure glucose levels are maintained at optimal levels (29).

Adverse events were reported more frequently in OHA-treated patients (12, 13). This is due to the known gastrointestinal side effects of OHAs (dizziness, nausea, vomiting, and fatigue), which could be exacerbated by dietary interventions (30). Furthermore, insulin-treated patients may have also experienced fewer adverse events due to the dose adjustments and closer clinical monitoring to avoid hypoglycemia. Additionally, the limited number of insulin-only studies (3, 18) available reduces the opportunity to detect and report adverse outcomes; thus, the findings should be interpreted cautiously.

Sulfonylureas pose a higher hypoglycemic risk than liraglutide, due to their promotion of continuous insulin secretion regardless of food intake (30). By contrast, liraglutide tends to be hypoglycemia-neutral, lowering hypoglycemia risk and improving glycemic control (31). However, GLP-1 agonists are commonly associated with gastrointestinal side effects, which may be exacerbated by fasting; prolonged periods without food can alter gastric emptying, modify drug absorption, and disrupt fluid balance, thereby increasing the severity of complications, such as the major single adverse event of gastroenteritis reported (30).

Compliance was high for fasting patients across all studies (3, 8, 10–14, 16, 17). The adherence rate was similar (~85%) for OHA and insulin-treated patients. This suggests that IF is a feasible long-term dietary approach for patients with T2DM (13, 16, 17). Furthermore, this emphasizes the safety of fasting for insulin-treated patients, usually perceived as being at higher risk when fasting (3), with careful monitoring and dosage adjustments.

IF is a highly practical intervention for OHA-treated patients. OHA-focused studies (8–10, 12–14) did not require constant monitoring of blood glucose or frequent dosage adjustment versus insulin treatment (3, 18), thereby reducing the burden of management.

### Strengths and limitations

Our findings here align with previous systematic reviews, suggesting that IF fasting strategies are beneficial in individuals with T2DM. Other comprehensive reviews (5, 6) have focused upon fasting interventions only, hence concluding that IF alone is not enough for T2DM treatment. Unlike prior studies that examined IF in isolation, our review demonstrates that the effects of fasting on glycemic control vary depending upon the pharmacological regimen, highlighting the role of IF as an adjuvant rather than a stand-alone therapy. This comparative approach fills an unexplored gap in the diabetes management literature.

Furthermore, minimizing heterogeneity in medication regimens (by excluding studies that included patients taking both medications simultaneously) allowed for a clearer determination of IF effects in OHA- versus insulin-treated patients.

By further sub-analysis of the effect of IF on different OHAs (metformin, sulfonylureas, and liraglutide), the potential confounding effect of grouping all OHAs was circumvented.

Methodologically, a strength of this review was the inclusion of RCTs only, ensuring high internal validity and minimization of bias. The generalizability of findings was strengthened by including studies involving ethnically diverse populations. Additionally, our evaluation of both efficacy and safety provides a balanced analysis of the therapeutic potential of IF in T2DM. Finally, the high adherence rate among studies demonstrates the feasibility of IF interventions in both insulin and OHAs-treated populations.

Limitations include the small number of studies available for inclusion, thus limiting the overall reliability and robustness of the review. While IF shows promising improvements in glycemic control and weight among patients with T2DM, the evidence base remains limited, particularly for insulin-treated individuals. Although insulin users appeared to experience a larger average HbA1c reduction (2.8%), this finding is derived from a minimal number of studies, with substantially smaller sample sizes compared with OHA-user cohorts. Moreover, study durations varied significantly, with 5 of the studies lasting only a few weeks (3, 8, 10, 16, 18), thereby providing limited insight into the sustainability and long-term safety of these metabolic improvements in insulin-treated patients. Therefore, despite encouraging initial results, the current evidence for insulin users should be interpreted with caution.

In terms of risk of bias assessment, high risk in multiple domains (allocation concealment, lack of participant blinding, and deviations from intended interventions) limits the reliability of the data and highlights the need for future studies to implement more rigorous risk mitigation strategies.

Another limitation was the substantial heterogeneity in the types of IF protocols used across the studies, which included TRF, ADF, FMD, and Ramadan fasting, as well as differences in fasting duration, caloric restriction, and fasting frequency; grouping all studies under a single “IF” umbrella without stratified analysis assumes homogeneity of physiological impact, which represents an inherent weakness. The heterogeneity in this review is driven by the differences in IF modalities and intervention durations. For instance, TRF primarily improves insulin sensitivity, whilst ADF and FMD induce deeper ketogenesis and greater caloric deficits, potentially producing larger reductions in weight and HbA1c (32). Ramadan fasting, however, involves prolonged daily fasting with nocturnal re-feeding, which attenuates metabolic benefits due to compensatory caloric intake and altered sleep patterns (15). As a result, pooling all interventions caused the variability in outcomes to increase.

## Conclusion

In conclusion, this review demonstrates that IF is beneficial in T2DM patients treated with both OHAs and insulin. Though there remains a need for more robust, high-quality, long-term RCTs to confirm and optimize the effect of IF, the current findings are still clinically relevant. They underscore the potential for medical healthcare professionals to consider IF as a conservative, cost-effective strategy for patient-centered management of T2DM. The benefits of fasting include improved glycemic control, increased insulin sensitivity, and reductions in adverse metabolic parameters in both insulin and OHA-treated patients. Here, we conclude that OHA-treated patients derive benefit with minimal risks. Similarly, insulin-treated patients derive benefits from fasting with few instances of hypoglycemia; however, caution and close monitoring are essential in this patient group during fasting periods.

As current evidence is limited by heterogeneity in IF protocols (type, duration, and baseline patient characteristics), future studies should focus on identifying the most effective regimes for T2DM patient subgroups.

## Data Availability

The original contributions presented in the study are included in the article/Supplementary material, further inquiries can be directed to the corresponding author.
