# Supplementary material for: Effects of intermittent fasting on HbA1c and weight in insulin versus oral hypoglycemic therapy-treated patients with type 2 diabetes mellitus: a systematic review and meta-analysis
Source: Front Nutr. 2026 Jan 30;13:1699384. doi: 10.3389/fnut.2026.1699384 (PMC12900757; doi:10.3389/fnut.2026.1699384)
Supplement: Supplementary file 1 [file Data_Sheet_1.PDF]

Appendices:

Supplementary table 1:

Database	Search Query
**PubMed**	#1 (Intermittent fasting) OR (time-restricted feeding) OR (periodic fasting) OR (cyclic fasting) OR (ADF) OR (prolonged fasting) OR (calorie restriction) OR (TRF) OR (fasting)
	#2 (Type 2 diabetes) OR (type 2 diabetes mellitus) OR (Adult onset diabetes) OR (maturity-Onset diabetes) OR (T2DM) OR (NIDDM) OR (non-insulin-dependent diabetes mellitus)
	#3 #1 AND #2
**Scopus**	(Intermittent fasting) OR (time-restricted feeding) OR (periodic fasting) OR (cyclic fasting) OR (ADF) OR (prolonged fasting) OR (calorie restriction) OR (TRF) OR (fasting) AND (Type 2 diabetes) OR (type 2 diabetes mellitus) OR (Adult onset diabetes) OR (maturity-Onset diabetes) OR (T2DM) OR (NIDDM) OR (non-insulin-dependent diabetes mellitus
**Cochrane Library**	(Intermittent fasting) OR (time-restricted feeding) OR (periodic fasting) OR (cyclic fasting) OR (ADF) OR (prolonged fasting) OR (calorie restriction) OR (TRF) OR (fasting) AND (Type 2 diabetes) OR (type 2 diabetes mellitus) OR (Adult onset diabetes) OR (maturity-Onset diabetes) OR (T2DM) OR (NIDDM) OR (non-insulin-dependent diabetes mellitus
